# Bile acids mediate liver-bone marrow crosstalk

**DOI:** 10.1016/j.isci.2026.116462

**Published:** 2026-06-23

**Authors:** Daniele Vitale, Mahmoud Karimi Azardaryany, Ghazal Alipour Talesh, Mahsa Shahidi, Vikki Ho, Suat Dervish, F.X. Himawan Haryanto Jong, Maito Suoh, Jacob George, Saeed Esmaili

**Affiliations:** 1 Storr Liver Centre, Westmead Institute for Medical Research, Westmead Hospital and University of Sydney, Westmead, NSW, Australia; 2 Westmead Research Hub, Westmead Institute for Medical Research, Westmead, NSW, Australia; 3 Department of Anatomy and Histology, Faculty of Medicine, Widya Mandala Catholic University of Surabaya, Surabaya, Indonesia; 4 Liver and Pancreatobiliary Diseases Research Centre, Digestive Disease Research Institute, Shariati Hospital, Tehran University of Medical Sciences, Tehran, Iran

**Keywords:** physiology, immunology, cancer

## Abstract

Disruption of liver metabolism alters immune responses, with maximal immune activation occurring when metabolic stress increases bone marrow hematopoietic stem cell (HSC) activity. We previously showed that sustained HSC activation enhances liver immune surveillance and reduces tumor burden. In the present study, we show that short-term HSC stimulation alone is insufficient for tumor control. Dietary supplementation with cholesterol and cholic acid, however, synergistically stimulates HSC responses and markedly elevates serum taurocholic acid (T-CA). Pharmacological inhibition of the apical sodium-dependent bile acid transporter (ASBT) restores bile acid homeostasis and lowers circulating T-CA, attenuating HSC activation and liver inflammation. Collectively, these findings reveal a bile acid-mediated liver-bone marrow crosstalk with direct implications for metabolic and neoplastic liver diseases.

## Highlights

• Cholesterol and cholic acid synergistically activate hematopoietic stem cells (HSCs)
• Taurocholic acid elevation associates with HSC expansion
• HSC responses parallel enhanced hepatic immune surveillance
• Sustained HSC engagement is required for liver tumor control

## Graphical abstract

**Figure:**
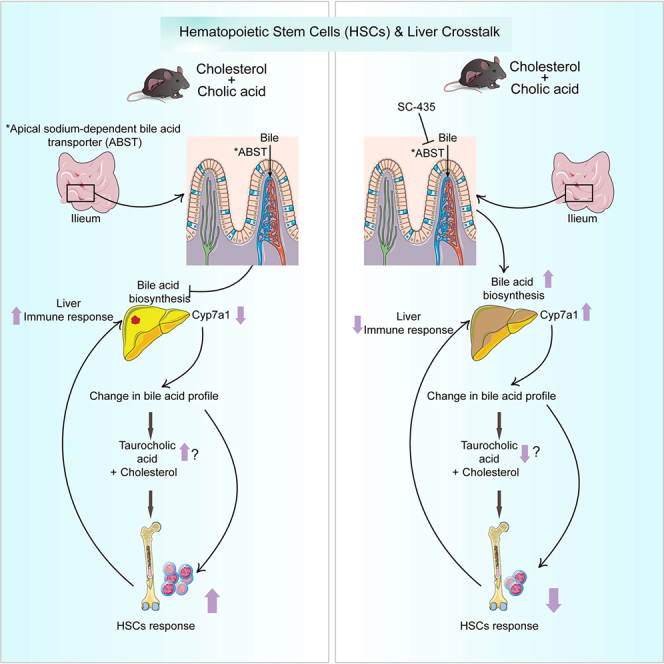

## Introduction

Liver exposure to nutrient overload and systemic metabolic stress alters both its metabolic and immune functions, increasing susceptibility to tumorigenesis.^1^ An exemplar is metabolic dysfunction-associated fatty liver disease (MAFLD), which is characterized by profound reprogramming of hepatic metabolic pathways and immune responses.^2^ Systems-level analyses of liver transcriptomes in human MAFLD and mouse models have revealed that the immune and metabolic related modules exhibit strong inverse correlations in their module behaviors (up-vs. downregulation).^2^ Notably, we showed that maximal immune responses in a mouse model coincide with activation of bone marrow hematopoietic stem cells (HSCs), marked by increased myeloid and lymphoid progenitor cells output.^3^ Sustained immune surveillance was associated with a reduced liver tumor burden, suggesting that inducing this state may represent a strategy to enhance anti-tumor immunity.^3^ Understanding the critical metabolic regulatory mechanisms that modulate distal hematopoietic responses therefore merits further study.

During embryonic development, the fetal liver serves as the primary site of hematopoiesis, supporting rapid expansion of HSCs before these cells colonize the bone marrow.^4^ Bile acids act as natural chemical chaperones that mitigate endoplasmic reticulum (ER) stress in expanding HSCs, supporting HSC growth by reducing protein aggregation and maintaining stem cell homeostasis.^4^ Emerging evidence suggests that bile acids continue to shape HSC biology in adulthood, influencing hematopoietic activity and differentiation beyond development.^5^^,^^6^ For example, the lysosomal handling of bile acids via transporters such as ENT3 influences intracellular bile acid availability and alleviates ER stress in HSCs, thereby supporting hematopoietic regeneration and erythropoiesis during stress recovery.^7^ Bile acid receptor signaling in the bone marrow microenvironment may also modulate niche components such as marrow adipose tissue, as has been reported in TGR5-deficient mice that exhibit altered stromal adipogenic profiles and changes in hematopoietic recovery capacity.^5^ This suggests that bile acids can modulate HSC responses in adulthood.

Bile acids are highly dynamic circulating metabolites that have been linked to changes in immune regulation and tissue homeostasis.^8^ Notably, a western-style high-fat/high-sucrose diet changes gut microbiota and bile acid profiles, altering the composition and concentrations of primary and secondary bile acids.^9^^,^^10^ Additionally, western-style and high-fat diets remodel the HSC transcriptional landscape, skewing differentiation toward pro-inflammatory myelopoiesis in models of obesity and metabolic disease, and establishing epigenetic and metabolic imprinting that contributes to systemic inflammation and immune dysfunction.^11^ Often, these studies use *Apoe*^−/−^ and *Ldlr*^−/−^ mice and western-style diets that can lead to HSCs expansion with a shift to myelopoiesis.^11^^,^^12^ Interestingly, hypercholesterolemia alone is not sufficient for inducing hematopoiesis in wild type mice transplanted with *Tet2*^−/−^ stem cells, while the presence of atherosclerosis trait complex is necessary.^12^ Aging similarly induces a shift toward myeloid bias at the expense of balanced multilineage output, contributing to decreased immune surveillance and greater susceptibility to disease. Restoring balanced hematopoiesis by antibody-mediated depletion of myeloid biased HSC improves immune responses to viral infection.^13^ In this study, we could induce balanced HSC activity in C57BL/6 mice fed a cholesterol-enriched diet supplemented with cholic acid (CA). Here, we investigate the effects of tumor presence, exposure time, dietary composition, and bile acid biosynthesis on shaping the bone marrow HSC response and crosstalk with the liver.

First, we demonstrate that the presence of liver tumors alone has minimal impact on HSC activation using mouse models of chemical carcinogenesis and tumor xenograft. We also tested whether short-term HSC stimulation was sufficient to control tumor growth. This showed that short-term activation per se is insufficient for tumor control, unlike long-term activation of HSCs (as we previously reported).^3^ When bile acid synthesis is perturbed through dietary manipulation, hematopoiesis is activated and systemic immune responses are heightened. Consistently, we detected an increase in serum taurocholic acid level in mice fed the cholesterol and CA diet. Restoration of bile acid homeostasis via pharmacological inhibition of ileal bile salt transport attenuated both HSC activation and liver immune responses. Expansion and contraction of the HSC population were associated with corresponding increases and decreases in taurocholic acid levels. Targeting this liver-bone marrow crosstalk may provide therapeutic opportunities in metabolic and neoplastic diseases.

## Results

### Limited HSC regulation by the presence of tumors

The hematopoietic system changes due to the release of immune modulators either by tumor cells or immune cells.^14^^,^^15^ Moreover, obesity and alterations in cholesterol metabolism can impact hematopoiesis, particularly when coupled with underlying genetic perturbations.^16^^,^^17^^,^^18^ We previously induced tumors using diethylnitrosamine (DEN), a well-established chemical model of liver carcinogenesis in mice that leads to progressive liver tumor formation.^3^ We reported that a prolonged and adequate bone marrow hematopoietic response in mice fed cholesterol and CA containing diets was associated with a reduction in liver tumor burden in wild-type mice. Notably, we observed only a weak HSC response in dietary models that included either sucrose alone or CA alone, and this response did not confer protection against tumor growth.^3^ It, however, remains unclear whether the presence of tumor in and of itself contributes to this weak HSC response.

**Figure 1:**
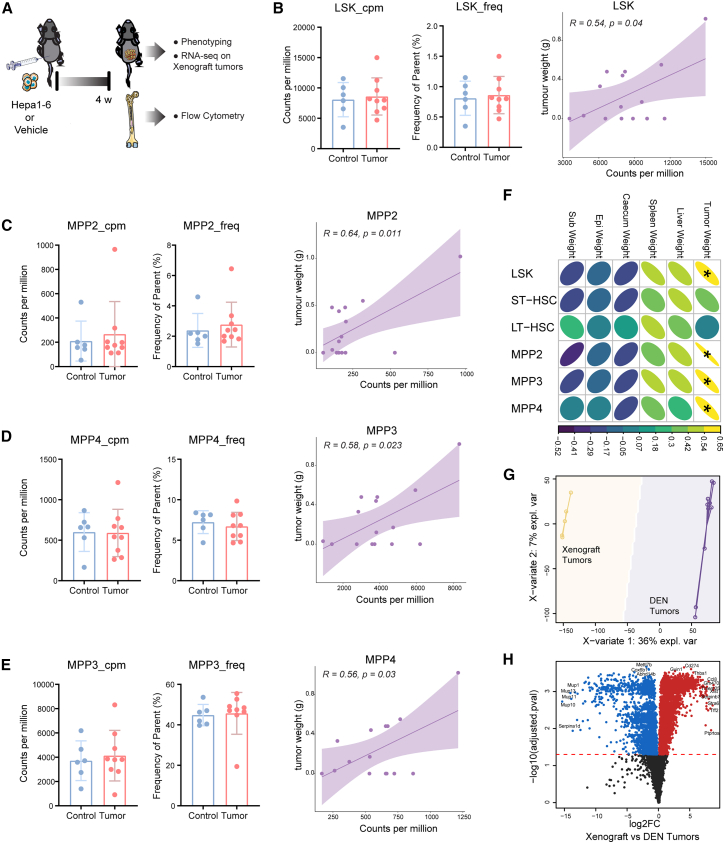
A positive association between tumor size and bone marrow HSPCs response in a xenograft tumor model (A) Schematic of xenograft tumor study with HSPCs immune profiling. (B–E) Flow cytometry analysis of bone marrow HSPCs and the associations with liver tumor size in control (*n* = 6) and tumor-bearing mice (*n* = 9). In bar charts, the data are indicated with mean ±1 standard deviation (SD). The counts and frequencies of each cell population were compared using two-tailed Mann-Whitney test. The association between the counts of HSPCs and tumor size were evaluated with Pearson’s correlation coefficients (*R*). The number of Lin^−^Sca1^+^c-kit^+^ (LSK) cells is positively correlated with tumor size (*R* = 0.54, *p* = 0.04). Analysis of LSK subpopulations demonstrates a positive correlation between tumor size and the number of MPP2 (*R* = 0.64, *p* = 0.011), MPP3 (*R* = 0.58, *p* = 0.023), or MPP4 (*R* = 0.56, *p* = 0.03). (F) Pairwise comparisons arranged in a matrix where the direction and magnitude of Pearson’s correlation between phenotypic variables is represented by the color of an ellipse whose pitch and eccentricity inform the direction and magnitude, respectively. Statistically significant correlations based on Pearson’s correlation coefficients are indicated (∗, *p* < 0.05). (G) PLS-DA analysis of RNA-seq in xenograft tumors (*n* = 5) and DEN (*n* = 10) induced tumors. Xenograft tumors are separated from DEN tumors. (H) Volcano plot shows large number of differentially expressed genes between xenograft and DEN induced tumors fed normal chow (NC) diet.

To address the issue, we used a xenograft tumor model by injecting Hepa 1-6 liver tumor cells into the flank of immunocompetent C57BL/6 mice. Tumors were harvested either 4 weeks later or when the estimated tumor weight reached 1 g; vehicle-injected mice served as controls (Figure 1A). We used flow cytometry to quantify bone marrow lineage-negative (Lin^−^) Sca-1^+^c-Kit^+^ (LSK) cells, which encompass hematopoietic stem and progenitor cells (HSPCs), including long-term and short-term HSCs (collectively referred to as HSCs) and multipotent progenitors 2, 3, and 4 (MPP2, MPP3, and MPP4) as previously described.^19^ This analysis revealed no differences in the number and frequencies of LSK cells or their subset progenitor populations between tumor-bearing mice and controls (Figures 1B–1E). However, we observed a positive correlation between tumor size and the number of LSK, MPP2, MPP3, and MPP4 cells (Figures 1B–1F). This suggests that very large tumors might regulate HSPC responses, either due to the presence of tumor or because the animals are stressed in the presence of large tumors, as previously demonstrated.^20^

To further investigate differences between tumor models, we performed RNA-seq on xenograft tumors and compared their transcriptomes with those of intrahepatic DEN-induced tumors from the normal chow (NC) group of a previous study.^3^ Partial least squares discriminant analysis (PLS-DA) and volcano plots of differentially expressed genes (DE genes) revealed distinct gene expression profiles between xenograft and DEN-induced tumors (Figures 1G and 1H). Because the differences in gene expression indicated that the xenograft model did not adequately recapitulate tumors that develop within the liver microenvironment, we focused subsequent studies on the DEN-induced tumor model.

### Short-term stimulation of the HSPCs’ response is insufficient to control tumor growth

**Figure 2:**
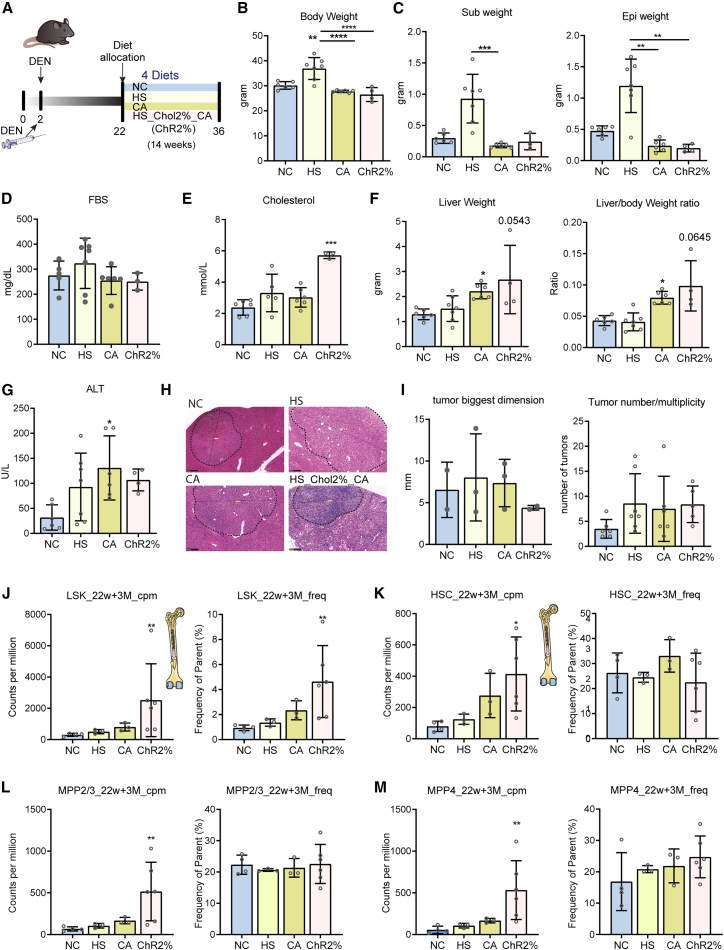
Short-term dietary challenge in a DEN tumor model did not show a synergism between diet and DEN tumors (A) Schematic shows the experimental plan with DEN injection (25 mg/kg) at the age of 2 weeks, 14 weeks of dietary challenge starting at 22 weeks of age (n = 5–7 mice/group). (B and C) HS diet increased mouse body weights as well as subcutaneous (sub) and epididymal (epi) adipose tissue weights (NC, *n* = 6; HS, *n* = 7; CA, *n* = 6; HS_Chol2%_CA, *n* = 5). (D) A higher trend in fasting blood glucose (FBS) in HS diet fed mice (NC, *n* = 5; HS, *n* = 7; CA, *n* = 6; HS_Chol2%_CA, *n* = 3). (E) Plasma cholesterol was highest in HS_Chol2%_CA diet fed mice (NC, *n* = 6; HS, *n* = 5; CA, *n* = 6; HS_Chol2%_CA, *n* = 3). (F) Liver weight and liver/body weight ratio were higher in CA and HS_Chol2%_CA diet fed mice (NC, *n* = 6; HS, *n* = 7; CA, *n* = 6; HS_Chol2%_CA, *n* = 5). (G) Higher alanine aminotransferase (ALT) levels in HS (a trend), CA (*p* < 0.05), and HS_Chol2%_CA (a trend) diet fed mice (NC, *n* = 6; HS, *n* = 7; CA, *n* = 6; HS_Chol2%_CA, *n* = 4). (H) Representative photomicrographs of H&E-stained liver sections from each diet group (original magnification, ×10; scale bar, 250 μm). (I) Similar tumor burden between HS_Chol2%_CA and NC diet fed mice (only animals with measurable tumors were included: NC, *n* = 2; HS, *n* = 3; CA, *n* = 3; HS_Chol2%_CA, *n* = 2). (J) Number and frequency of Lin^−^Sca-1^+^c-Kit^+^ cells (LSKs) increased in HS_Chol2%_CA diet group, with a higher trend in CA diet fed mice (NC, *n* = 4; HS, *n* = 3; CA, *n* = 3; HS_Chol2%_CA, *n* = 6). (K) The number and frequencies of hematopoietic stem cells (HSCs) (long-term and short-term HSCs) between diet groups. (L and M) Higher number of multipotent progenitor cells (MPPs) 2/3 (L) and MPP4 (M) in HS_Chol2%_CA diet group. Error bars represent mean ± 1 standard deviation (SD). One-way ANOVA followed by Tukey’s HSD post-hoc test (parametric data) or the Kruskal–Wallis test followed by Dunn’s post-hoc test (non-parametric data) were used for statistical analysis (∗∗∗∗*p* < 0.0001, ∗∗∗*p* < 0.001, ∗∗*p* < 0.01, ∗*p* < 0.05). NC, normal chow; HS, high sucrose; CA, cholic acid; HS_Chol2%_CA, high sucrose + high cholesterol (2%) + cholic acid.

Previously, we used long-term dietary exposure to study tumor behavior over a 36-week study. This approach elicited different degrees of HSPC responses, which may have contributed to divergent tumor outcomes between the diet groups.^3^ Here, we tested whether reducing the duration of diet exposure could differentially affect HSC responses and tumor outcomes. Because prolonged stimulation by diet can also lead to HSC exhaustion, we investigated whether a shorter, high-intensity induction of HSC responses would confer improved protective outcomes. Thus, we subjected 22-week-old mice to 14 weeks of diets that varied in their composition. We used high-sucrose (HS), NC with CA 0.5% (CA), or HS_Chol2%_CA (a cholesterol rich diet with CA) diet challenges, while control mice were DEN-injected and fed NC diet (Figure 2A).

Mice exposed to the HS diet had higher body weights and adipose tissue mass and exhibited a trend toward higher blood glucose levels (Figures 2B–2D). Mice fed the HS_Chol2%_CA diet had higher blood cholesterol levels (Figure 2E). Both CA and HS_Chol2%_CA diets increased liver weight and liver/body weight ratio (Figure 2F). Plasma alanine aminotransferase (ALT) levels were elevated in the HS, CA (*p* < 0.05), and in the HS_Chol2%_CA diet compared to control mice (Figure 2G). These findings suggest that dietary composition differentially impacts metabolic and liver-related parameters in the context of DEN-induced tumorigenesis. Notably, we did not detect significant differences in liver tumor burden between mouse groups exposed to these dietary interventions (Figures 2H and 2I) suggesting that chronic dietary exposure, as reported previously,^3^ is required to elicit differential tumor growth.

Importantly, flow cytometry of HSPCs from the mice revealed no response to an HS diet (compared to NC diet) and a trend toward an elevated HSC response in mice fed the CA diet. On the other hand, long-term exposure to the HS diet elicited a subtle HSC response alongside increased tumor burden in our previous study.^3^ In contrast, the HS_Chol2%_CA diet group exhibited significantly higher numbers and frequencies of LSK cells (Figure 2J), and the numbers of HSC, MPP2/3, and MPP4 cells (Figures 2K–2M), although no differences were observed in the frequencies of their subpopulations (Figures 2K–2M). The MPP2/3 are myeloid biased cells and generate all myeloid lineages, while MPP4 cells are biased toward lymphoid cells.^21^^,^^22^ Notably, short-term exposure to cholesterol and CA stimulated an HSPC response but did not alter tumor burden, indicating that long-term stimulation of HSPC is required for a protective effect.

### Bile acid biosynthesis modulates bone marrow HSPC responses

**Figure 3:**
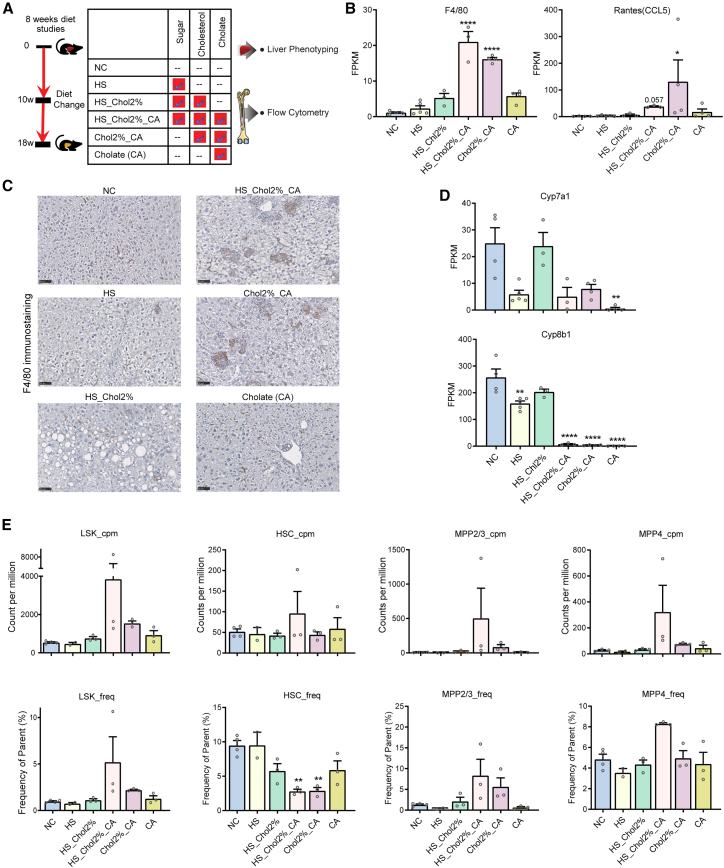
Dietary challenge in mice induced a spectrum of liver pathologies and bone marrow hematopoiesis responses (A) Schematics show the composition of 6 diets. (B) Liver inflammatory markers are upregulated in diets containing cholesterol and cholic acid (NC, *n* = 4; HS, *n* = 5; HS_Chol2%, *n* = 3; HS_Chol2%_CA, *n* = 3; Chol2%_CA, *n* = 4; CA, *n* = 4). (C) Representative photomicrographs of liver F4/80 immunostaining showing increased macrophage infiltration in livers of mice fed cholesterol- and cholic acid-containing diets (original magnification ×40; scale bar, 50 μm). (D) Suppression of bile acid biosynthesis in mice exposed to cholic acid (NC, *n* = 4; HS, *n* = 5; HS_Chol2%, *n* = 3; HS_Chol2%_CA, *n* = 4; Chol2%_CA, *n* = 4; CA, *n* = 4). (E) Higher number of Lin^−^Sca-1^+^c-Kit^+^ (LSK) cells in mice fed the diet containing cholesterol and cholic acid (NC, *n* = 4; HS, *n* = 2; HS_Chol2%, *n* = 3; HS_Chol2%_CA, *n* = 3; Chol2%_CA, *n* = 3; CA, *n* = 3). Error bars represent mean ± 1 standard deviation (SD). One-way ANOVA followed by Tukey’s HSD post-hoc test (parametric data) or the Kruskal–Wallis test followed by Dunn’s post-hoc test (non-parametric data) were used for statistical analysis (∗∗∗∗*p* < 0.0001, ∗∗∗*p* < 0.001, ∗∗*p* < 0.01, ∗*p* < 0.05).

Our current and previous DEN tumor studies show that diet exposure time is an important factor in modulating the HSPC response.^3^ To define the minimal requirements for HSPC stimulation, we examined HSPC changes in previous non-tumor dietary studies.^2^ The diets encompassed different combinations of sucrose and cholesterol, with and without CA, namely: (A) NC; (B) HS; (C) high-sucrose + high cholesterol (2%) (HS_Chol2%); (D) HS + high cholesterol 2% + CA 0.5% (HS_Chol2%_CA); (E) high cholesterol 2% + CA 0.5% (Chol2%_CA); and (F) CA 0.5% (CA) (Figure 3A).

The expression of the macrophage marker F4/80 and the inflammatory marker Rantes within the liver was upregulated in diets that contained both CA and cholesterol: HS_Chol2%_CA, and Chol2%_CA (Figure 3B). Notably, no significant difference was observed between HS_Chol2%_CA, and Chol2%_CA diets, indicating a synergistic effect of CA with cholesterol. We noticed a higher trend in the expression of macrophage markers in the HS_Chol2%, and CA diet fed mice (Figure 3B). Immunostaining for F4/80 confirmed significant accumulation of macrophages in diets containing both cholesterol and CA (Figure 3C). The HS_Chol2% diet showed a degree of immune infiltration, while the high-sucrose diet alone failed to induce inflammation. Examining liver metabolic responses to dietary challenge showed that the expression of genes related to bile acid synthesis such as *Cyp7a1* and *Cyp8b1* was consistently and significantly lower in mice exposed to diets with added CA (Figure 3D). Exposure to sucrose also reduced the expression of these markers (Figure 3D). High-sucrose diets have been reported to affect bile acid synthesis and metabolism.^23^ This modulation of bile acid homeostasis with long-term sucrose exposure may have contributed to the weak HSPC response we observed previously.^3^

Investigation of bone marrow HSCs via flow cytometry analysis of mice on these diets revealed a trend toward increased number and percentage of LSKs or HSPCs in diets enriched in cholesterol and CA (Figure 3E). Importantly, the HS_Chol2% diet (without CA) and CA diet did not have a significant impact on the bone marrow-HSPCs response (Figure 3E). Overall, these findings highlight the essential roles of specific components, particularly cholesterol and CA, in driving HSPC responses.

Further analysis of the HSPC sub-populations indicated an increasing trend in MPP2, MPP3, and MPP4 in diets enriched in cholesterol and CA. The number of HSCs (long-term and short-term HSCs) was not different between diets, although their frequencies were decreased in diets that contained cholesterol and CA (Figure 3E). This decrease in HSC frequency can be attributed to the fact that the majority of LSKs (the parent cells utilized to calculate HSC frequencies) in diets that contained cholesterol and CA were of the multipotent progenitor cell population. Our findings suggest a systemic response and overall activation of bone marrow HSCs as well as their differentiation into progenitor cells in diets that contain both cholesterol and CA (Figure 3E).

**Figure 4:**
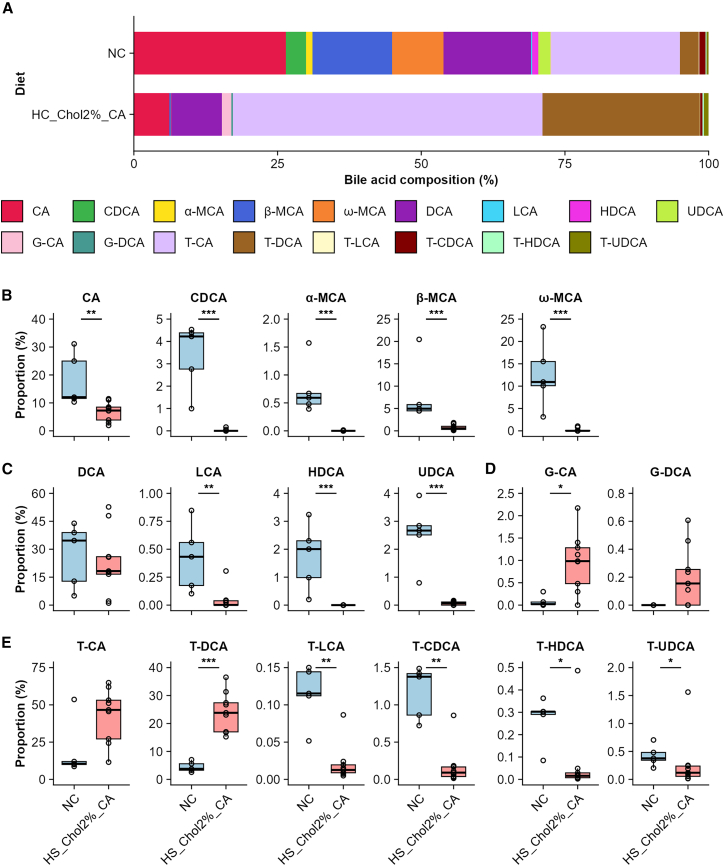
The HS_Chol2%_CA diet causes a dramatic shift toward taurine-conjugated bile acids such as taurocholic acid (T-CA), with higher serum levels of the secondary bile acid deoxycholic acid (DCA), while cholic acid levels are reduced (A) The bile acid composition between mice on NC or HS_Chol2%_CA diet. (B–E) The proportion of primary bile acids (B), secondary bile acids (C), glycine-conjugated bile acids (D), and taurine-conjugated bile acids (E) to the total bile acids in mice on NC or HS_Chol2%_CA diet. In each boxplot, the bottom, middle, or upper line indicates first quartile, median, or third quartile, respectively. The whiskers extend no greater than 1.5 times the interquartile range, and all data are shown as points. Two-tailed Mann-Whitney test was used for statistical comparison between NC and HS_Chol2%_CA diet groups (∗∗∗∗*p* < 0.0001, ∗∗∗*p* < 0.001, ∗∗*p* < 0.01, ∗*p* < 0.05) (NC, normal chow, *n* = 5; HS_Chol2%_CA, high sucrose + high cholesterol (2%) + cholic acid, *n* = 9). CA, cholic acid; CDCA, chenodeoxycholic acid; DCA, deoxycholic acid; G-CA, glycocholic acid; G-DCA, glycodeoxycholic acid; HDCA, hyodeoxycholic acid; LCA, lithocholic acid; T-CA, taurocholic acid; T-DCA, taurodeoxycholic acid; T-LCA, taurolithocholic acid; T-UDCA, tauroursodeoxycholic acid; T-CDCA, taurochenodeoxycholic acid; T-HDCA, taurohyodeoxycholic acid; UDCA, ursodeoxycholic acid; α-MCA, alpha-muricholic acid; β-MCA, beta-muricholic acid; ω-MCA, omega-muricholic acid.

Shortening the duration of dietary exposure showed that feeding mice the HS_Chol2%_CA diet consistently activates HSC responses. To further understand the underlying mechanisms, we measured serum bile acid levels to assess changes in bile acid profiles. Interestingly, CA levels were reduced in the HS_Chol2%_CA group, whereas its byproducts taurocholic acid (T-CA), taurodeoxycholic acid (T-DCA), glycocholic acid (G-CA), and glycodeoxycholic acid (G-DCA) levels were increased (Figures 4A–4E). Notably, T-CA has been shown to support HSC growth *in vitro* and promote HSC expansion in the fetal liver.^4^ Thus, the increase in circulating T-CA may have contributed to the observed expansion of HSCs in the HS_Chol2%_CA diet group.

**Figure 5:**
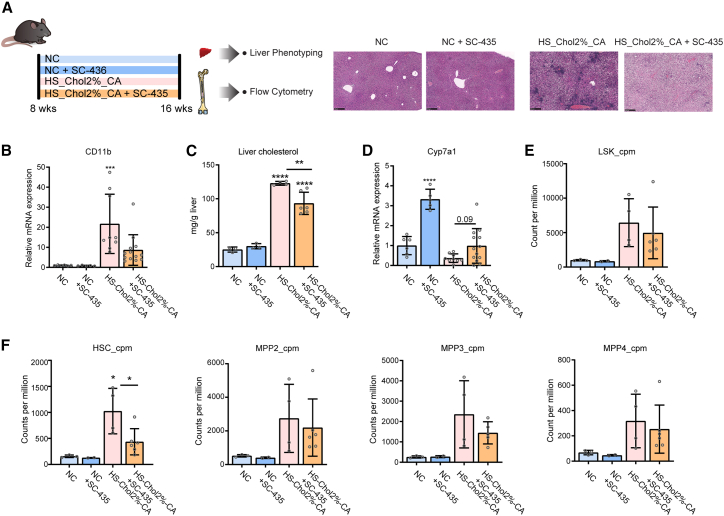
Regulating bile acids metabolism can regulate bone marrow HSPCs response (A) Experimental design and liver histology of HS_Chol2%_CA diet study with or without SC-435 (original magnification ×10; scale bar, 250 μm). (B) Relative mRNA expression of *CD11b* shows SC-435 treatment alleviated liver immune cell infiltration (NC, *n* = 6; NC + SC-435, *n* = 5; HS_Chol2%_CA, *n* = 9; HS_Chol2%_CA + SC-435, *n* = 13). (C) Mice fed the HS_Chol2%_CA diet showed increased total liver cholesterol while adding SC-435 slightly reduced total liver cholesterol (NC, *n* = 3; NC + SC-435, *n* = 3; HS_Chol2%_CA, *n* = 4; HS_Chol2%_CA + SC-435, *n* = 6). (D) SC-435 increased liver *Cyp7a1* mRNA expression (NC, *n* = 6; NC + SC-435, *n* = 5; HS_Chol2%_CA, *n* = 9; HS_Chol2%_CA + SC-435, *n* = 13). (E) Feeding mice the HS_Chol2%_CA diet increased Lin^−^Sca-1^+^c-Kit^+^ (LSK) cell numbers in the bone marrow, while SC-435 treatment showed a trend toward reducing the LSK response. (F) The analysis of bone marrow hematopoietic stem and progenitor cell (HSPC) subpopulations (LSK cells) shows reduction in the number of hematopoietic stem cells (HSCs) with SC-435 treatment in mice on HS_Chol2%_CA diet (NC, *n* = 3; NC + SC-435, *n* = 2; HS_Chol2%_CA, *n* = 4; HS_Chol2%_CA + SC-435, *n* = 6). See also Figure S1. Error bars represent mean ± 1 standard deviation (SD). One-way ANOVA followed by Tukey’s HSD post-hoc test (parametric data) or the Kruskal–Wallis test followed by Dunn’s post-hoc test (non-parametric data) were used for statistical analysis (∗∗∗∗*p* < 0.0001, ∗∗∗*p* < 0.001, ∗∗*p* < 0.01, ∗*p* < 0.05).

We previously reported that treatment with SC-435, a selective inhibitor of the apical sodium-dependent bile acid transporter (ASBT), alters bile acid composition, characterized by increased CA and a marked reduction in serum T-CA levels.^24^ Here, we further analyzed the cytometry data on bone marrow HSPC populations from mice in that study (Figure 5A). In this model, detailed quantification of liver injury and inflammatory markers has been reported^24^ and phenotypic data in Figures 5A–5D are provided as contextual information. The decreasing trend in *CD11b* mRNA expression indicates a reduction in myeloid cell infiltration in the livers of mice fed the HS_Chol2%_CA diet and treated with SC-435 (Figure 5B). SC-435 treatment also resulted in a reduction in liver cholesterol levels (*p* < 0.01), although levels remained substantially elevated compared with normal controls (Figure 5C). Moreover, SC-435 enhanced hepatic *Cyp7a1* mRNA expression, a key marker of bile acid biosynthesis, in the NC group. Although *Cyp7a1* expression was suppressed in mice fed the HS_Chol2%_CA diet, SC-435 normalized *Cyp7a1* expression and effectively reversed the inhibitory effect of CA (Figure 5D).

Analysis of bone marrow flow cytometry data revealed a reduction in the number of HSCs (*p* < 0.05) in HS_Chol2%_CA diet-fed mice treated with SC-435, but not in LSKs which consists of HSCs and MPPs (Figures 5E, 5F, and S1). This reduction in HSC numbers indicates that biosynthesis of bile acids as measured using *Cyp7a1* mRNA expression, and plausibly a change in serum bile acid profile with reduction of taurocholic acid (as we reported previously in these mice^24^) may have a role in reducing the stimulatory effects on HSCs expansion. Although the number of bone marrow progenitor cells remained high, the immediate impact on liver immune responses was attenuated.

## Discussion

We previously reported that metabolic perturbations in the liver lead to increased hepatic immune activity that is associated with elevated bone marrow HSPC activity. However, the key hepatic drivers of this liver-bone marrow cross-talk remained unclear. In the present study, we investigated how dietary composition, exposure duration, and tumor burden influence the liver-bone marrow interaction. Using a xenograft tumor model, we found that only very large tumors could elicit an HSC response, making it unlikely that smaller intrahepatic tumors drive this effect. Shortening exposure to an HS_Chol2%_CA diet in a DEN-induced liver tumor model was sufficient to activate HSPCs but did not reduce tumor burden, indicating that the duration of stimulation is critical for differential tumor outcomes. Further studies in a non-tumor model revealed that in mice fed cholesterol and CA, serum levels of CA were reduced while levels of its byproduct taurocholic acid were increased. Previous work has shown that taurocholic acid preferentially supports HSCs expansion over the broader Lin^−^ and LSK populations, both *in vitro* and *in vivo*.^4^ We previously found that restoring bile acid synthesis using the ASBT inhibitor SC-435 reduced serum taurocholic acid levels. Here, we show that this reduction is associated with decreased HSC numbers but does not affect progenitor cells, identifying HSCs as the primary population regulated by bile acids in our model system.

Time is a critical determinant of biological and therapeutic outcomes, particularly in immunometabolic regulation. Using comparable dietary interventions in tumor-bearing mice with differing durations of exposure, we demonstrated that short-term increases in bone marrow HSC activity do not alter tumor outcomes, whereas long-term, sustained liver immune responses are required to achieve differential tumor effects.^3^ This provides important evidence that modulation of bile acid signaling to stimulate HSPCs does not induce overt tolerance or exhaustion of the HSPC compartment. Although the functional efficacy of differentiated immune cells to maintain immune surveillance may decline over time, HSCs themselves exhibited a sustained response, suggesting a degree of resilience within the stem cell pool. This is consistent with findings that bile acids cause expansion of HSCs in fetal liver and protects them from ER stress and the unfolded protein response.^4^ Interestingly, a recent analysis of patients in the IMbrave150 clinical trial (immunotherapy in hepatocellular carcinoma) showed that responders had downregulation in pathways related to cholesterol homeostasis and bile acid synthesis.^25^ Consistent with the broader concept that HSC and MPP engagement drives anti-tumor responses, Priem et al.^26^ developed a bone-marrow-targeted nanobiologic therapy, MTP_10_-HDL (HDL-based nanodiscs), that induces trained immunity by engaging HSCs and MPPs *in vivo*. Treatment with MTP_10_-HDL suppressed tumor growth and enhanced checkpoint inhibitor efficacy. The anti-tumor effect was preserved when bone marrow from treated donors was transplanted into naive recipients, demonstrating that the therapeutic benefit derives from durable reprogramming of the HSC/MPP compartment.

We observed that SC-435 mediated modulation of bile acid synthesis appears to have a greater impact on HSCs, with only modest changes in the heterogeneous LSK compartment, which comprises both HSCs and MPPs. Consistent with this SC-435 reduced phenotypic HSCs numbers while largely sparing MPPs, resulting in an attenuated effect when analyzing the broader LSK population. While our sample size may not have provided sufficient power to detect all differences among HSPC subpopulations, the consistent HSC responses observed across independent experiments support the biological relevance of the reported phenotype. Notably, we previously reported that levels of DCA and T-DCA remain elevated in the SC-435-treated group.^24^ Previous studies have shown that secondary bile acids such as DCA can directly act on myeloid progenitors via the vitamin D receptor (VDR) to promote myelopoiesis.^6^ While bile acid receptors such as FXR and TGR5 are minimally expressed in HSCs,^6^ they are expressed in components of the bone marrow niche, suggesting that bile acids may regulate HSC homeostasis indirectly through niche-mediated mechanisms.^5^

Collectively, our data suggest that HSPC activation occurs largely independently of tumor presence, that short-term diet-based HSPC activation is insufficient for tumor control, and that sustained HSPCs activity may represent a promising immunomodulatory strategy for neoplastic liver disease. The role of taurocholic acid in this process—expanding HSC populations while protecting them from stress—is of interest and warrants further investigation.

### Limitations of the study

This study was conducted exclusively in male mice, and the findings may not capture sex-specific differences in bile acid metabolism or hematopoietic responses, given that estrogen signaling modulates both processes. The translational relevance of the identified liver-bone marrow crosstalk in humans also remains to be established, although the observation that immunotherapy responders in the IMbrave150 trial display downregulation of cholesterol and bile acid pathways is consistent with our findings.

Although we observed associations between circulating taurocholic acid levels and HSC expansion, our data do not establish direct causality. Additional studies using controlled bile acid supplementation or receptor-specific genetic models will be required to determine whether taurocholic acid directly and preferentially regulates HSC activity relative to broader progenitor populations. Furthermore, in the SC-435 study, HSPC subpopulation analyses may have been underpowered to detect differences across all progenitor compartments. The contribution of HSC activation to liver immune surveillance also warrants more formal evaluation using transplantation or lineage-tracing approaches.

Finally, our experiments were performed in young and middle-aged mice with relatively responsive HSC compartments; the findings may therefore not fully reflect HSC biology in aged settings, where stem cell exhaustion, myeloid bias, and clonal hematopoiesis become prevalent. Future longitudinal studies incorporating serial sampling will be needed to define how aging and bile acid profiles jointly influence hematopoiesis and anti-tumor immunity.

## Resource availability

### Lead contact

Further information and requests for resources should be directed to and will be fulfilled by the lead contact, Saeed Esmaili (saeed.esmaili@sydney.edu.au).

### Materials availability

The study did not generate new materials.

### Data and code availability

• Data: Mouse liver RNA-seq data from the xenograft tumor study have been newly generated for this paper and deposited at NCBI GEO; they are publicly available as of the date of publication under accession number GSE325236. Mouse liver RNA-seq data from the six-diet study were previously deposited under accession number GSE325237 and reported in Esmaili et al. (2021; https://doi.org/10.1016/j.cels.2021.04.004). Mouse flow cytometry data, phenotypic measurements, qPCR readouts, and bile acid concentrations underlying the figures have been deposited at Mendeley Data and are publicly available as of the date of publication. Owing to dataset size limits on a single Mendeley repository, flow cytometry data are split across two deposits: flow cytometry data from the six-diet experiment are available under DOI: https://doi.org/10.17632/sfng2h249n.3 (previously released dataset), and all other flow cytometry data together with phenotypic, qPCR, and bile acid source data are available under DOI: https://doi.org/10.17632/xxmyn33ynr.1. Accession numbers and DOIs are listed in the key resources table.
• Code: All original R code used in this study — for processing flow cytometry data, performing RNA-seq differential expression and gene-set enrichment analyses, and analyzing bile acid composition — has been deposited at Mendeley Data and is publicly available as of the date of publication under DOI: https://doi.org/10.17632/xxmyn33ynr.1. The DOI is also listed in the key resources table.
• Other items: Any additional information required to reanalyze the data reported in this paper is available from the lead contact upon request.

## Acknowledgments

S.E. and J.G. are supported by the Robert W. Storr Bequest to the Sydney Medical Foundation, University of Sydney; The Snow Program for Liver Health, Snow Medical Research Foundation; a 10.13039/501100000925National Health and Medical Research Council of Australia (10.13039/501100000925NHMRC) program grant (APP1149976); project grants (APP1107178 and APP1145766) and an Investigator Grant (APP1196492). The mouse tumor studies were supported by the 10.13039/501100007811Sydney Medical School Foundation. G.A.T. and D.V. are supported by scholarships from the University of Sydney. We thank Sally Coulter for performing the bile acid measurements and for providing technical assistance with the analyses. Animal studies, flow cytometry, histological analyses, and RNA-seq experiments were performed at the Westmead Scientific Platforms, which are supported by the Westmead Research Hub, the Westmead Institute for Medical Research, the 10.13039/501100001171Cancer Institute New South Wales, the 10.13039/501100025520Medical Research Future Fund, the 10.13039/501100000925National Health and Medical Research Council, and the 10.13039/501100001047Ian Potter Foundation.

## Author contributions

Conceptualization, S.E.; methodology, S.E., M.K.A., and D.V.; project administration, S.E., M.K.A., and D.V.; resources, S.E. and J.G.; investigation, D.V., M.K.A., G.A.T., V.H., S.D., F.X.H.H.J., M.S., and S.E.; formal analysis, D.V.; visualization, D.V. and M.S.; data curation, S.E.; writing – original draft, S.E. and D.V.; writing – review and editing, M.S., S.E., and J.G.; funding acquisition, J.G. and S.E.; supervision, S.E. and J.G.

## Declaration of interests

The authors declare no competing interests.

## Declaration of generative AI and AI-assisted technologies in the writing process

During the preparation of this work, the authors used ChatGPT (OpenAI) for language editing, and Claude (Anthropic) to assist with the organization of the deposited R analysis code. After using these tools, the authors reviewed and edited the content as needed and take full responsibility for the content of the publication.

## STAR★Methods

### Key resources table

**Table:** 

REAGENT or RESOURCE	SOURCE	IDENTIFIER
**Antibodies**		

Rat anti-mouse F4/80 (clone Cl:A3-1)	Bio-Rad	Cat#MCA497
Goat anti-rabbit IgG (H+L) Secondary Antibody, Biotin	Thermo Fisher Scientific	Cat#31820; RRID: AB_228340
APC rat anti-mouse CD117	BD Biosciences	Cat#553356; RRID: AB_398536
PE-CF594 rat anti-mouse CD140A	BD Biosciences	Cat#562775; RRID: AB_2737786
BUV737 rat anti-mouse CD16/CD32	BD Biosciences	Cat#565272; RRID: AB_2739145
Brilliant Violet 510™ rat anti-mouse CD127 (IL-7Rα)	BioLegend	Cat#135033; RRID: AB_2564576
Brilliant Violet 650™ rat anti-mouse CD150 (SLAM)	BioLegend	Cat#115932; RRID: AB_2715765
Brilliant Violet 785™ rat anti-mouse CD90.2 (Thy1.2)	BioLegend	Cat#105331; RRID: AB_2562900
Brilliant Violet 711™ rat anti-mouse Ly-6A/E (Sca-1)	BioLegend	Cat#108131; RRID: AB_2562241
APC/Cyanine7 rat anti-mouse CD48 Antibody	BioLegend	Cat#103432; RRID: AB_2561463
BV421 rat anti-mouse CD34	BD Biosciences	Cat#562608; RRID: AB_11154576
BUV395 rat anti-mouse CD45	BD Biosciences	Cat#564279; RRID: AB_2651134
FITC Armenian hamster anti-mouse/rat CD29	BioLegend	Cat#102205; RRID: AB_312882
PE/Cyanine7 rat anti-mouse CD105	BioLegend	Cat#120410; RRID: AB_1027700
PE rat anti-mouse CD135	BD Biosciences	Cat#553842; RRID: AB_395079
Brilliant Violet 650™ rat anti-mouse/human CD44	BioLegend	Cat#103049; RRID: AB_2562600

**Chemicals, peptides, and recombinant proteins**		

Matrigel® Growth Factor Reduced (GFR) Basement Membrane Matrix, LDEV-free	Corning	Cat#354230
Diethylnitrosamine	Sigma-Aldrich	Cat#73861
SC-435	Lumena/Shire Pharmaceuticals	N/A
tert-Butyl alcohol	Sigma-Aldrich	Cat#360538
Triton X-100	Sigma-Aldrich	Cat#X100
Methanol	Sigma-Aldrich	Cat#E7023
Rodent Decloaker, 10X	Biocare Medical	Cat#RD913
BLOXALL Endogenous Blocking Solution, Peroxidase and Alkaline Phosphatase	Vector Laboratories	Cat#SP-6000-100
Protein Block, Serum-Free	Dako	Cat#X0909
Antibody Diluent, Background Reducing	Dako	Cat#S3022
Streptavidin, Peroxidase	Vector Laboratories	Cat#SA-5004-1
ImmPACT® DAB Substrate, Peroxidase (HRP)	Vector Laboratories	Cat#SK-4105
Ammonium Chloride Solution	STEMCELL Technologies	Cat#7850
Zombie Yellow™ Fixable Viability Kit	BioLegend	Cat#423103
Brilliant Stain Buffer	BD Biosciences	Cat#566349
RNase-Free DNase Set	Qiagen	Cat#79256

**Critical commercial assays**		

Cholesterol E	Wako	Cat#439-17501
FavorPrep™ Tissue Total RNA Extraction Mini Kit	FAVORGEN	Cat#FATRK001
Truseq stranded mRNA library prep kit	Illumina	Cat#20020595

**Deposited data**		

Liver RNA-seq, mouse (xenograft tumor study)	This paper / NCBI GEO	GSE325236
Liver RNA-seq, mouse (six-diet study)	Esmaili et al., 2021 / NCBI GEO	
Mouse flow cytometry, phenotypic, qPCR, and bile acid source data	This paper / Mendeley Data	https://doi.org/10.17632/xxmyn33ynr.1
Mouse flow cytometry data (six-diet experiment)	This paper / Mendeley Data	https://doi.org/10.17632/sfng2h249n.3
Original R analysis code	This paper / Mendeley Data	https://doi.org/10.17632/xxmyn33ynr.1

**Experimental models: Cell lines**		

Hepa 1-6	ATCC	Cat#CRL-1830

**Experimental models: Organisms/strains**		

Male C57BL/6 mice	Animal Resources Centre	C57BL/6JArc

**Oligonucleotides**		

Primer: Cyp7a1 Forward: GGAGCTTATTTCAAATGATCAGG Reverse: CACTCTGTAAAGCTCCACTCACTT	This paper	N/A

**Software and algorithms**		

GraphPad Prism	Dotmatics	https://www.graphpad.com/
R 4.0.3	R Core Team^27^	https://www.r-project.org/
RStudio 1.2.5033	Posit Software^28^	https://posit.co/download/rstudio-desktop/
ggcyto 1.18.0	Van et al.^29^	https://www.bioconductor.org/packages/release/bioc/html/ggcyto.html
openCyto 2.2.0	Finak et al.^30^	https://www.bioconductor.org/packages/release/bioc/html/openCyto.html
flowStats 4.2.0	Hahne et al.^31^	https://www.bioconductor.org/packages/release/bioc/html/flowStats.html
flowCore 2.2.0	Hahne et al.^32^	https://www.bioconductor.org/packages/release/bioc/html/flowCore.html
CATALYST 1.14.0	Nowicka et al.^33^	https://www.bioconductor.org/packages/release/bioc/html/CATALYST.html
FlowSOM 1.22.0	Van Gassen et al.^34^	https://bioconductor.org/packages/release/bioc/html/FlowSOM.html
ConsensusClusterPlus 1.54.0	Wilkerson & Hayes^35^	https://bioconductor.org/packages/release/bioc/html/ConsensusClusterPlus.html
diffcyt 1.10.0	Weber et al.^36^	https://bioconductor.org/packages/release/bioc/html/diffcyt.html
ggplot2 3.3.3	Wickham^37^	https://ggplot2.tidyverse.org/index.html
glmm 1.4.2	Knudson et al.^38^	https://cran.r-project.org/web/packages/glmm/index.html
multcomp 1.4-17	Hothorn et al.^39^	https://cran.r-project.org/web/packages/multcomp/index.html
Trimmomatic 0.39	Bolger et al.^40^	https://github.com/usadellab/Trimmomatic
Salmon 1.4.0	Patro et al.^41^	https://combine-lab.github.io/salmon/
tximeta 1.8.2	Love et al.^42^	https://github.com/thelovelab/tximeta
DESeq2 1.30.1	Love et al.^43^	https://bioconductor.org/packages/release/bioc/html/DESeq2.html
mixOmics 6.14.0	Rohart et al.^44^	https://bioconductor.org/packages/release/bioc/html/mixOmics.html
NOISeq 2.34.0	Tarazona et al.^45^	https://bioconductor.org/packages/release/bioc/html/NOISeq.html
edgeR 3.32.1	Chen et al.^46^	https://bioconductor.org/packages/release/bioc/html/edgeR.html
limma 3.46.0	Ritchie et al.^47^	https://bioconductor.org/packages/release/bioc/html/limma.html
EDASeq 2.24.0	Risso et al.^48^	https://bioconductor.org/packages/release/bioc/html/EDASeq.html
corrplot 0.84	Wei & Simko^49^	https://cran.r-project.org/web/packages/corrplot/
PMCMRplus 1.6.0	Pohlert^50^	https://cran.r-project.org/web/packages/PMCMRplus/

**Other**		

Meat Free Rat and Mouse Diet (Normal Chow)	Specialty Feed	N/A
High Sucrose diet	Specialty Feed	SF09-079
High Sucrose + Cholesterol (2%) diet	Specialty Feed	SF14-158
High Sucrose + Cholesterol (2%) + Cholic acid (0.5%) diet	Specialty Feed	SF09-080
Meat free Rat and mouse diet + Cholesterol (2%) + Cholic acid (0.5%) diet	Specialty Feed	SF14-007
Meat free Rat and mouse + Cholic acid (0.5%) diet	Specialty Feed	SF11-105

### Experimental model and study participant details

#### Mice

All procedures were approved by the Western Sydney Local Health District Animal Ethics Committee (WSLHD AEC) under protocol numbers 4228.10.14 and 4246.10.15 and conducted in accordance with the Animal Experimentation guidelines of the National Health and Medical Research Council (NHMRC) of Australia. Male C57BL/6 mice were obtained from Animal Resources Centre (Perth, Australia) and maintained in Westmead Bioresources Facility of The Westmead Institute for Medical Research. The mice were exposed to a 12-hour light/dark cycle in specific pathogen-free conditions with free access to food and water.

In xenograft tumor model, Hepa 1-6 cells (mouse hepatoma cell line) were cultured in 1X Dulbecco's Modified Eagle Medium (DMEM) supplemented with 10% fetal bovine serum (FBS), 1% penicillin-streptomycin (Sigma-Aldrich) and 1X GlutaMAX (Thermo Fisher Scientific) at 37°C and 5% CO_2_. The cell suspension of Hepa 1-6 was prepared at a concentration of 1 × 10^7^ cells/mL and mixed at 1:1 ratio with growth factor-reduced Matrigel (Corning, Cat# 354230) to yield a final concentration of 5 × 10^6^ cells/mL. A volume of 200 μL (containing 1 × 10^6^ cells) was slowly injected into each flank in mice aged 6 to 8 weeks old under isoflurane anesthesia. Tumors were harvested either 4 weeks later or when the estimated tumor weight reached 1 g.

For the chemical-induced liver cancer study, male mice were injected intraperitoneally with 25 mg/kg body weight DEN (Sigma-Aldrich, Munich, Germany) at 14 days of age. These mice were then given the diets containing 34% sucrose (HS diet), cholic acid (0.5%) added diet (CA), or a cholesterol rich diet which contained high sucrose (34%), 2% cholesterol and 0.5% cholic acid (HS_Chol2%_CA) (Specialty Feed Service, Glen Forest, Australia) starting from week 22 for 14 weeks. The control group was fed with the normal chow (NC) diet throughout the experiment. The mice were harvested at 34 weeks after injection (at the age of 36 weeks).

In diet studies without liver cancer development, mice were given the NC, HS, HS_Chol2%, HS_Chol2%_CA, Chol2%_CA, or CA (0.5%) diets for 8 weeks starting at 8–10 weeks of age. In pharmacologic interventional experiments, mice were fed with NC or HS_Chol2%_CA diets with/out SC-435 for 8 weeks starting at 8 –10 weeks of age, as we reported previously.^24^

At the end of each study, mice were euthanized by intraperitoneal injection of ketamine (100 mg/kg body weight) (Mavlab, Australia) and xylazine (10 mg/kg body weight) (Troy Laboratories, Australia).

### Method details

#### Biochemical assays

Following euthanasia, mouse blood was collected by cardiac puncture and stored at −80°C until use. Serum ALT were measured by automated techniques within the Department of Clinical Chemistry, Westmead Hospital. Serum lipid panel was measured using Cobas b 101 POC system and its lipid disc (Roche Diagnostics). For liver cholesterol measurements, total lipids were extracted using the method described by Shimabukuro et al.^51^ Briefly, 30 mg of frozen liver was used to extract total lipids with 3:2 tert-butyl alcohol:triton X-100/methyl alcohol (1:1). Liver cholesterol levels were determined by enzymatic colorimetric assays using Wako E Total Cholesterol Kit (Wako, Cat# 439-17501) according to the manufacturer's protocol.

#### Histology

The harvested mouse liver and tumor tissues were fixed with 10% neutral buffered formalin for 24 hours. The liver histology was assessed on 5μm paraffin embedded sections stained with hematoxylin and eosin (H&E).

#### Immunostaining

Heat-induced epitope retrieval was applied on formalin fixed paraffin embedded (FFPE) liver tissue sections (4 μm) with 1x Rodent Decloaker buffer (Biocare Medical, Cat# RD913) at 95°C for 40 minutes. Sections were incubated with peroxidase blocking solution (Bloxall, Vector Laboratories, Cat# SP-6000-100) for 10 minutes. After washing in TBST, slides were incubated with DAKO Protein Block, Serum-Free (DAKO, Cat# X0909) for 45 minutes, followed by overnight incubation with F4/80 antibody (Clone CI:A3-1, Bio-Rad Laboratories) diluted in DAKO antibody diluent (1:100) with background reducing components (DAKO, Cat# S3022). For DAB staining, a biotin-labeled secondary antibody (goat anti-rabbit) (Invitrogen, Cat# 31820), 1:500 diluted in DAKO antibody diluent was incubated for 1 hour at room temperature. Subsequently, slides were washed in TBST and incubated with Streptavidin HRP (Vector Laboratories, Cat# SA-5004-1) for 10 minutes at room temperature. ImmPACT DAB Substrate (Vector Laboratories, Cat# SK-4105) was used for developing and counterstained with Mayer’s Hematoxylin for 1 minute.

#### Bone marrow cell isolation

Femurs were removed from mice using forceps and surgical scissors, cleaned of muscle and connective tissue and placed in wells of 6-well plates containing 3 mL of cold DPBS. 0.5 mL tubes were pierced using a 21G syringe and inserted into 1.5 mL tubes. Bone ends were cut using a scalpel and centrifuged by fast ramp up to 12,000 × *g* to extract the bone marrow. All centrifugations were done at 4°C. Bone marrow pellets were resuspended in 400 μL of IMDM + 2% FBS then passed through a 70 μm strainer, flushing through with 10 mL of the medium into a 50 mL tube. Cells were pelleted at 300 × *g* for 5 minutes and RBC lysed using Ammonium Chloride Lysis Buffer (STEMCELL Technologies, Cat# 7850) as per manufacturer protocol. Cells were washed twice in IMDM + 2% FBS and kept on ice.

#### Flow cytometry

Prepared cell pellets were suspended in 1000 μL FACS buffer (DPBS containing 5% heat-inactivated FBS and 5 mM EDTA). The live singlets were counted using DAPI 1:10 dilution by mixing 45 μL of cell suspension with 5 μL DAPI by gating on FSC-A vs FSC-H measuring average event/μL on CytoFlex (Beckman Coulter). Subsequently, 10 million bone marrow cells/sample were transferred to round bottom 96 well plates and pelleted by spinning at 500 × *g* for 5 minutes. Cell viability assessed by incubation with 50 μL Zombie Yellow (BioLegend, Cat# 423103) viability stain (1:500 in DPBS) for 15 minutes in the dark, at room temperature. The cells were then topped up with 200 μL DPBS, spun down at 500 × *g* for 5 minutes at 4°C. The supernatant was gently aspirated, and the pellet suspended in 100 μL of antibody cocktail in Brilliant stain buffer (BD Biosciences, Cat# 566349) for 30 minutes in the dark and at room temperature. The cells were topped with 200 μL FACS buffer, spun down at 500 × *g* for 5 min at 4°C. The supernatant was then gently aspirated, and the pellet was washed 3 times with 200 μL FACS buffer and spun down at 500 × *g* for 5 minutes at 4°C. After the final wash, the pellet was suspended in 300 μL FACS buffer and transferred to a FACS tube and kept on ice in the dark. Fluorometric data were then acquired on a flow cytometer (Fortessa, Becton Dickinson). One well of unstained cells was included in all experiments. Single color controls (SCCs) were prepared by adding only one single antibody to the cells in FACS buffer. Fluorescence minus one (FMO) controls were prepared by adding all the antibodies of each panel except for one to the FACS buffer. A pooled mixture of all samples was used for unstained wells, SCCs and FMOs.

We used the following antibodies for flow cytometry: CD117 APC 2B8 (BD, Cat# 553356), CD140A PE-CF594 APA5 (BD, Cat# 562775), CD16/CD32 BUV737 2.4G2 (BD, Cat# 565272), Brilliant Violet 510 anti-mouse CD127 (IL-7Rα) (BioLegend, Cat# 135033), Brilliant Violet 650 anti-mouse CD150 (SLAM) (BioLegend, Cat# 115932), Brilliant Violet 785 anti-mouse CD90.2 (BioLegend, Cat# 105331), Brilliant Violet 711 anti-mouse Ly-6A/E (Sca-1) (BioLegend, Cat# 108131), APC/Cy7 anti-mouse CD48 (BioLegend, Cat# 103432), Anti-mouse CD34 BV421 (BD, Cat# 562608), Anti-mouse CD45 BUV395 (BD, Cat# 564279), FITC Anti-mouse/rat CD29 (BioLegend, Cat# 102205), PE/Cy7 anti-mouse CD105 (BioLegend, Cat# 120410), PE Rat Anti-Mouse CD135 (BD, Cat# 553842), Brilliant Violet 650 anti-mouse/human CD44 Antibody (BioLegend, Cat# 103049).

Raw FCS files were compensated, arcsinh transformed (a = 0, b = 1/150, c = 0) cleaned (margin events, debris, singlets, live) and cell populations of interest (Lin^−^Sca-1^+^c-Kit^+^ [LSK], Lin^−^Sca-1^-^c-Kit^+^ [LKS^-^] and residual Lin^−^) were gated using functions provided by ggcyto_1.18.0,^29^ openCyto_2.2.0,^30^ flowStats_4.2.0^31^ and flowCore_2.2.0.^32^ CATALYST_1.14.0^33^ provided wrapper functions for FlowSOM^34^ and ConsensusClusterPlus. Pre-gated LSK, LKS^−^, and residual Lin^−^ cells were clustered based on cell marker expression (fluorescence intensity) (Figure S2). Similar clusters were manually merged into cell subtypes of interest. Plots were generated using ggplot2_3.3.3.^37^ The glm function provided by glmm_1.4.2^38^ was used to fit linear models (family = gaussian) where cells/million live singlets of studied populations and diet were the response and independent variables, respectively. Pairwise adjusted *p* values were generated using Tukey's honest significance test using the glht function provided by multcomp_1.4-17.^39^ The analyses were performed in R 4.0.3^27^ (R Core Team) global environment using RStudio IDE 1.2.5033^28^ (Posit Software).

#### Gene expression analysis

The excised mouse liver and liver tumor tissues were snap frozen in liquid nitrogen and stored at −80°C. Total RNA from liver or tumor tissues was extracted using Favor Prep Tissue Total RNA Extraction Mini Kit (FAVORGEN, Cat# FATRK001) with DNase I treatment (Qiagen, Cat# 79256) according to the manufacturer's instructions and stored at −80°C until use. RNA concentration was measured using Nanodrop spectrophotometer (Thermo Fisher). Gene expression was measured by qPCR or by RNA-seq for tumor xenografts, as we reported recently.^3^

The purity and integrity of total RNA from liver or tumor tissues were confirmed using TapeStation (Agilent). The RNA-seq libraries were prepared from 100 ng total RNA using Truseq stranded mRNA library prep kit (Illumina, Cat# 20020595). The library average fragment length and standard deviation (SD) was verified using TapeStation (Agilent) before 100 bp SE sequencing on the NovaSeq 6000 (Illumina), yielding a median of 20 million reads per sample in mouse tumor study. Sequencing adapters and low-quality reads were trimmed using the ‘Maximum information quality filtering’ algorithm implemented in Trimmomatic-0.39.^40^ A cut-off length of 40 and strictness setting 0.999 was used.^40^ The cut-off for the minimum read length was set to 36 bp.

Reads were mapped using Salmon-1.4.0^41^ against the GENCODE vM25 mouse transcriptome with GRCm38 mouse genome assembly as a decoy. The Salmon mapping algorithm was used with both random hexamer priming and GC bias correction.

The R package tximeta-1.8.2^42^ was used to generate raw count matrices from Salmon quant.sf output files. Reads were summarized at the gene level and raw count matrices exported as csv for downstream processing. The PLS-DA between DEN-induced and xenograft liver tumors was implemented with plsda function in mixOmics package.^44^ For differential gene expression analysis between DEN-induced and xenograft liver tumor models, the volcano plot was created with NOISeq package,^45^ with significant genes colored at a cut-off of adjusted *p* value below 0.05.

#### Bile acids measurement

The measurement of bile acids from mouse serum samples was performed in a high-throughput manner using Ultra-Performance Liquid Chromatography (UPLC; Shimadzu, Kyoto, Japan), ACQUITY (Waters, Milford, MA) column, and Q-TRAP 5500 Mass Spectrometer (AB SCIEX, Toronto, Canada) as previously described.^24^ Calibration curves were generated using standards containing 23 bile acids, prepared across a range of concentrations in pooled naïve plasma rendered bile acid-free by activated charcoal treatment. The limit of detection for individual bile acids was 0.01–0.05 μmol/L.

Individual bile acids were classified as primary, secondary, glycine-conjugated, or taurine-conjugated, and per-class proportions were computed as the sum of class concentrations divided by the total bile acid concentration per animal. Differences between the NC and HS_Chol2%_CA diet groups were tested using two-tailed Mann–Whitney U tests.

### Quantification and statistical analysis

In mouse phenotypic data and flow cytometry analysis of tumor and diet studies, the results are shown in bar charts with mean ± 1 standard deviation (SD). The comparisons between two groups were performed with a two-tailed Student’s t test (parametric data) or Mann–Whitney U test (non-parametric data). The comparisons among three or more groups used one-way ANOVA followed by Tukey’s HSD post-hoc test (parametric data) or the Kruskal–Wallis test followed by Dunn’s post-hoc test (non-parametric data). Pearson's correlation coefficients (*R*) and the *p* values were used to examine the association between two continuous variables. (∗∗∗∗*p* < 0.0001, ∗∗∗*p* < 0.001, ∗∗*p* < 0.01, ∗*p* < 0.05).

The results of bile acid analysis are presented as Tukey boxplots. Each box indicates the first quartile, median, and third quartile, with the whiskers extending no greater than 1.5 times the interquartile range. The difference between the proportion of each bile acid was compared with two-tailed Mann–Whitney tests.

Sample sizes in each mouse studies are indicated in the figure legends. No statistical test was used to predetermine the sample size, and mice were randomly allocated to the experimental groups. Statistical analyses were performed using GraphPad Prism (Dotmatics) or R 4.0.3^27^ (R Core Team) with RStudio 1.2.5033.^28^
