# Supplementary material for: Bile acids mediate liver-bone marrow crosstalk
Source: iScience. 2026 Jun 23;29(7):116462. doi: 10.1016/j.isci.2026.116462 (PMC13320317; doi:10.1016/j.isci.2026.116462)
Supplement: Document S1. Figures S1 and S2 [file mmc1.pdf]

iScience, Volume *29*

Supplemental information

Bile acids mediate liver-bone marrow crosstalk

Daniele Vitale, Mahmoud Karimi Azardaryany, Ghazal Alipour Talesh, Mahsa Shahidi, Vikki Ho, Suat Dervish, F.X. Himawan Haryanto Jong, Maito Suoh, Jacob George, and Saeed Esmaili

Supplementary Figure

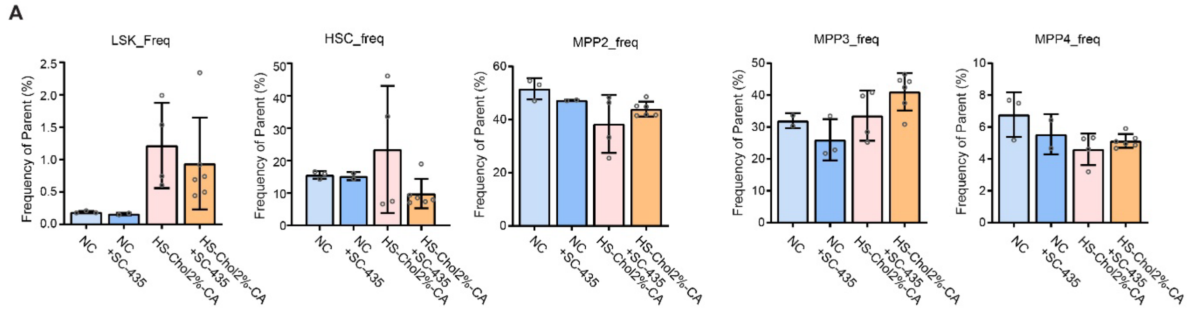

Figure S1. Frequencies of LSK cells and their subpopulations in the mouse diet and SC-435 study, Related to Figure 5. Feeding mice the HS_Chol2%_CA diet increased the frequency of Lin$^-$Sca-1$^+$c-Kit$^+$ (LSK) cells, while SC-435 treatment showed no significant effect on the frequencies of LSK cells or their subpopulations. (NC, n = 3; NC + SC-435, n = 2; HS_Chol2%_CA, n = 4; HS_Chol2%_CA + SC-435, n = 6). Error bars represent mean ±1 standard deviation (SD). One-way ANOVA was used to test for differences in means with Tukey's (parametric) and Dunn's (non-parametric) multiple comparison post-hoc test (****$p < 0.0001$, ***$p < 0.001$, **$p < 0.01$, and *$p < 0.05$).

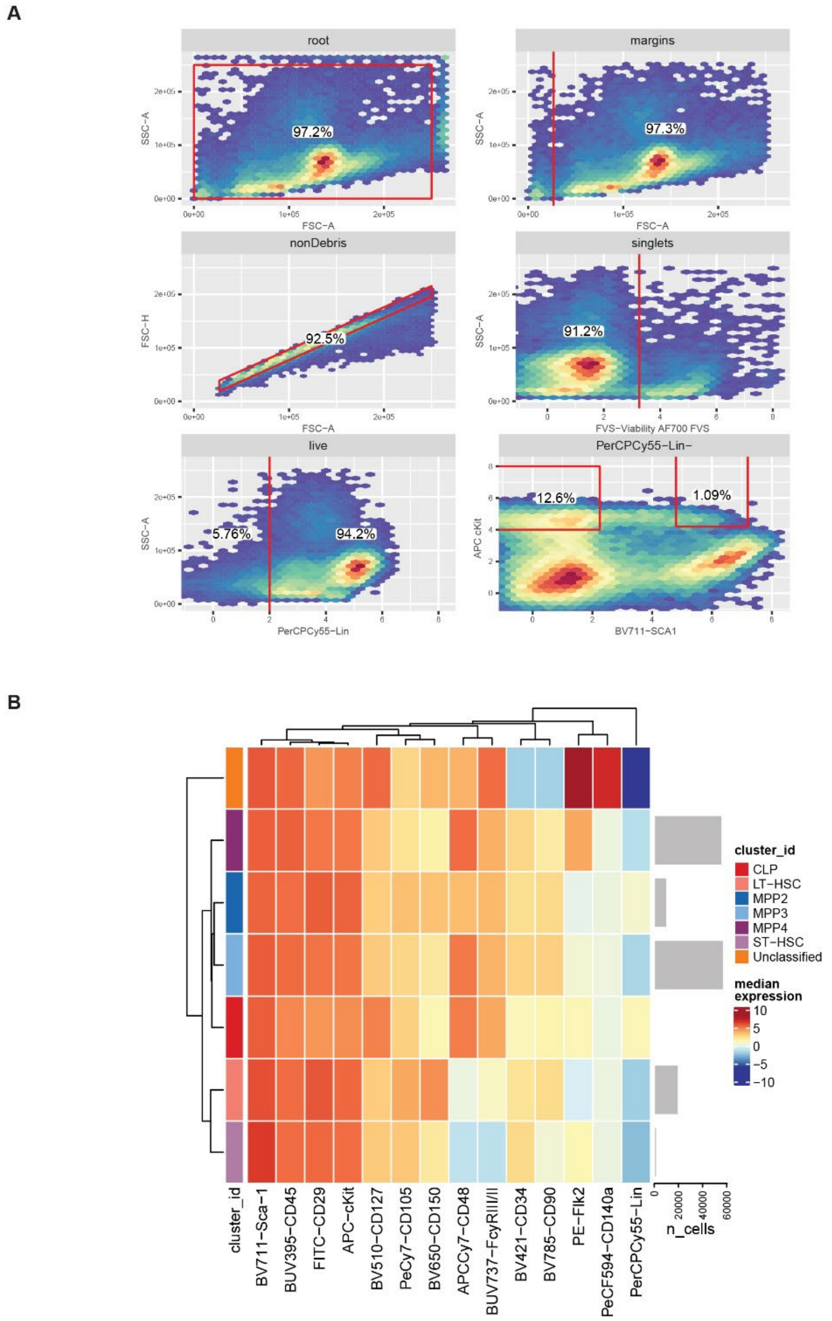

Figure S2. Flow cytometry gating strategy and clustering, Related to STAR Methods. A) Flow-cytometry gating strategy to identify hematopoietic stem and progenitor cells population (Lin$^-$Sca-1$^+$c-Kit$^+$). B) Subsequently, we performed clustering to identify hematopoietic stem cells (defined as the combined population of long-term HSCs and short-term HSCs), as well as multipotent progenitors (MPPs).
